# Strengths and weaknesses of the acute care systems in the United Kingdom and the Netherlands: what can we learn from each other?

**DOI:** 10.1186/s12873-019-0257-y

**Published:** 2019-07-26

**Authors:** Marjolein N. T. Kremers, Prabath W. B. Nanayakkara, Marcel Levi, Derek Bell, Harm R. Haak

**Affiliations:** 1 Department of Health Services Research, and CAPHRI School for Public Health and Primary Care, Aging and Long Term Care, Maastricht University, Maastricht, the Netherlands; 2 Department of Internal Medicine, Máxima Medical Centre, Postbox 90052, 5600 PD, Veldhoven/Eindhoven, the Netherlands; 3 Section Acute Medicine, Department of Internal Medicine, Amsterdam Public Health research institute, Amsterdam UMC, location VUmc, Amsterdam, the Netherlands; 4 Department of Medicine, University College London Hospitals NHS Foundation Trust, London, UK; 5 NIHR CLAHRC Northwest London, Imperial College London, Chelsea and Westminster Hospital, Fulham Road, London, SW10 9NH UK; 6 Department of Internal Medicine, Division of General Internal Medicine, Maastricht University Medical Centre+, Maastricht, the Netherlands

**Keywords:** Emergency care, Organisation of care, Health care quality

## Abstract

**Background:**

The demand on Emergency Departments and acute medical services is increasing internationally, creating pressure on health systems and negatively influencing the quality of delivered care. Visible consequences of the increased demand on acute services is crowding and queuing. This manifests as delays in the Emergency Departments, adverse clinical outcomes and poor patient experience.

**Overview:**

Despite the similarities in the UK’s and Dutch health care systems, such as universal health coverage, there are differences in the number of patients presenting at the Emergency Departments and the burden of crowding between these countries. Given the similarities in funding, this paper explores the similarities and differences in the organisational structure of acute care in the UK and the Netherlands. In the Netherlands, less patients are seen at the ED than in England and the admission rate is higher. GPs and so-called GP-posts serve 24/7 as gatekeepers in acute care, but EDs are heterogeneously organised. In the UK, the acute care system has a number of different access points and the accessibility of GPs seems to be suboptimal. Acute ambulatory care may relieve the pressure from EDs and Acute Medical Units. In both countries the ageing population leads to a changing case mix at the ED with an increased amount of multimorbid patients with polypharmacy, requiring generalistic and multidisciplinary care.

**Conclusion:**

The acute and emergency care in the Netherlands and the UK face similar challenges. We believe that each system has strengths that the other can learn from. The Netherlands may benefit from an acute ambulatory care system and the UK by optimizing the accessibility of GPs 24/7 and improving signposting for urgent care services. In both countries the changing case mix at the ED needs doctors who are superspecialists instead of subspecialists. Finally, to improve the organisation of health care, doctors need to be visible medical leaders and participate in the organisation of care.

## Background

The demand on Emergency Departments (ED) and acute medical services is increasing internationally, [1, 2] creating pressure on health systems and negatively influencing the quality of delivered care. [3] Demographic changes and governmental policy changes play an important role in this increasing demand. [4] A direct association between an aging population and increased utilization of emergency services exists. [5] In addition, medical patients presenting at the ED are often characterized by multimorbidity and polypharmacy leading to complex clinical presentations needing more diagnostics and multidisciplinary care. [6]

Visible consequences of the increased demand on acute services are crowding and queuing: a situation wherein the need for emergency services exceeds available resources at the ED or in the hospital. [7] This manifests as delays in the EDs, adverse clinical outcomes and poor patient experience. [3, 8] Factors that influence crowding across Europe are an ageing population, improved treatment modalities, limited human and physical hospital resources and delayed ancillary services. [9]

Despite the similarities in the UK’s and Dutch health care system, such as universal health coverage, there are differences in the number of patients presenting at the EDs and the burden of crowding between these countries. There are 0.54 EDs per 100,000 people in the Netherlands, compared to 0.33 in England. [10] The amount of available hospital beds per capita in the Netherlands is 2.4/1,000 (in 2015) and 2.6/1,000 in the UK (in 2016). [11, 12] Both countries have a comparable level of prosperity and healthcare is funded by a mix of private and public payments. The UK spent 9.9% of their Gross Domestic Product (GDP) on healthcare in 2015, whereas the Netherlands 10.6% of its GDP in the same year (Table 1). [13] Given the similarities in funding, this paper explores the similarities and differences in the organisational structures of the acute care systems in these countries, focussing on the acute medical (non-trauma) care, and discuss potential lessons. In addition, we will suggest directions for a future-proof organisation of acute medical care based on integrating the strengths of both systems.

**Table 1:** Numbers and properties of the Dutch and British acute care systems in 2016

	The Netherlands	England
Number of EDs per 100,000 people	0.54^c^	0.33
Hospital beds per 1,000 people	2.4^d^	2.6
Percentage of GDP spent on healthcare	10.6^d^	9.9^d^
Available GPs per 10,000 people	5.8	7.6^b^
Number of ED visits per year	2,400,000	15,900,000^a^
ED attendance rate	14.1	24.2^a^
Number of acute admissions per year	840,000	4,300,000
Acute admission rate	4.9	6.6
Percentage of acute admissions for the total ED visits	35.0	27.0^a^

^a^Data based on type 1 and 2 Emergency Departments only

^b^ Data retrieved over 2013

^c^ Data retrieved over 2014

^d^ Data retrieved over 2015

### The Dutch system

**Fig. 1:**
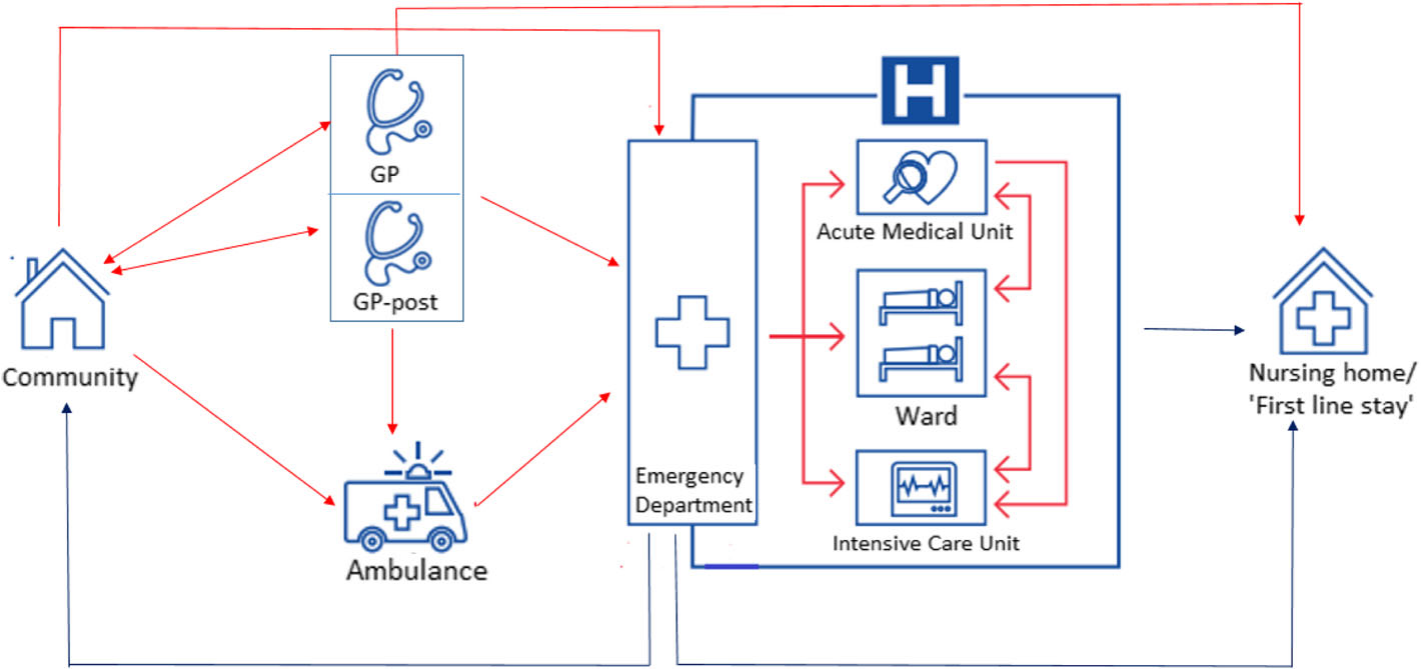
The acute care chain in the Netherlands. (Adapted with permission from design by LS van Galen for her thesis “Patient Safety in the Acute Healthcare Chain: is it safer@home?”)

Acute care in the Netherlands is mainly provided by general practitioners (GP) and via EDs (Fig. 1). GPs take care of patients with urgent primary care needs, while EDs provide care for patients who urgently need specialized care. There were 5.8 GPs available for a population of 10,000 in 2016. [14] To gain access to hospital care, including EDs, patients are required to have a referral from a GP or directly transferred by an ambulance. However, some patients still attend the ED directly, despite the fact that patients have to pay an initial deductible for self-referral to the ED. Interestingly, the introduction of the deductible resulted in a substantial reduction of self-referrals. Care provided by the GP or out-of-hours GP services is covered by compulsory health insurance without an initial deductible.

During out-of-hours, GPs mostly cooperate to provide urgent primary care on rotation basis, taking care of each other’s patients in so-called GP-posts. This ensures the gatekeeping function of the GP 24/7. A GP-post can be reached out-of-hours by phone, upon which a nurse under supervision of a GP will carry out triage using the Dutch Triage Standard. [15]

In 2016 in the Netherlands 2.4 million ED visits took place for a population of 17 million. This means an attendance rate of 14%. 840,000 patients were admitted, which is 35% of all patients visiting the ED and 4.9% of the population. [1] Fifty-six percent of the patients were referred to the ED via a GP and 23% was self-referred. The remaining 21% presented at the ED by ambulances via emergency calls. [1] One organisational innovation to improve inappropriate use of the ED is a collaboration between GPs and EDs: an Emergency Care Access Point (ECAP). GPs and EDs both have their own departments, while sharing the same entrance and joint triage by a nurse. In this situation, 75% of the self-referred patients are seen by a GP, which is safe and cost-effective. [16] However, ECAPs are only present in 22% of all Dutch EDs. [1]

Instead of referring a patient to the ED, GPs can also refer patients needing admission due to medical or social reasons, but not in need for specialized care, to a so called ‘first line stay’. This is a medical institution runned by GPs or elderly care physicians, providing care for a maximum duration of 3 months. These ‘first line stays’ may prevent unnecessary ED visits, especially in elderly patients, which is needed for a sustainable acute care system taking the increased demand of ED services by patients > 65 years into account. Since the 1st of April 2018 regional coordination points for first line care have been introduced, aiming for more efficient bed management by providing 24/7 insight in available beds. However, in September 2018 GPs mentioned that only in 21% of all cases they were able to find a first line bed on the same day of presentation. On top of that, GPs still experience difficulties in obtaining information about the available beds, especially during out-of-hours shifts. [17] As a consequence, many low-complex patients are still being admitted to the hospital via the ED. Of all patients > 65 years presenting at the ED in 2017, 17% could have received the needed care at a first line stay facility. [18]

In the Netherlands, the staffing of the EDs is heterogeneously organized: emergency physicians are not present in every hospital, nevertheless their role as coordinators of care in many EDs is increasing. Historically, residents of different medical specialties staff the ED in collaboration with residents in emergency medicine. They are fully qualified doctors who either are in training to become specialists or non-trainees who are working in the hospitals to gain experience with the aim of entering a specialist training programme at a later date. Residents are remotely supervised by consultants, such as internists and surgeons. Only since 2009 emergency medicine was recognized as a specialty. While acute physicians are increasingly present at the ED, consultants from other specialties are rarely present at the ED. The quite inexperienced residents in these specialities are taking care of the patients with complex problems. Although ED physicians can see these patients initially and stabilize them, multidisciplinary teams with more specific expertise are needed to treat complex patients presenting at the ED.

During the last few years there’s a slight decrease in the total number of ED visits, but there has been a 14% increase in ED visits by patients > 65 years between 2013 and 2016. However, the percentage of people > 65 years in the population increased from 16,8% in 2013 to 18,2% in 2016, which is only an 8% increase. [1, 19] The number of admissions from the ED increased from 33,2% in 2013 to 35,5% in 2016. [1] The governmental policy changes have forced elderly patients to stay at home longer, leading to reduced surveillance. A simple problem in these patients therefore may go undetected for a few days leading to complex presentations.

From the ED, patients can be admitted to an Acute Medical Unit (AMU), in general for up to 72 h, or a medical ward. AMUs in the Netherlands are often used by medical as well as surgical specialities. Although possible, it is uncommon to admit patients directly from the outpatient department at the AMU.

### The British system

In the UK the National Health Service (NHS) is responsible for providing acute and emergency care. The organisation of the acute care chain differs between the four UK nations in terms of structure, but from a patient perspective is broadly similar.

**Fig. 2:**
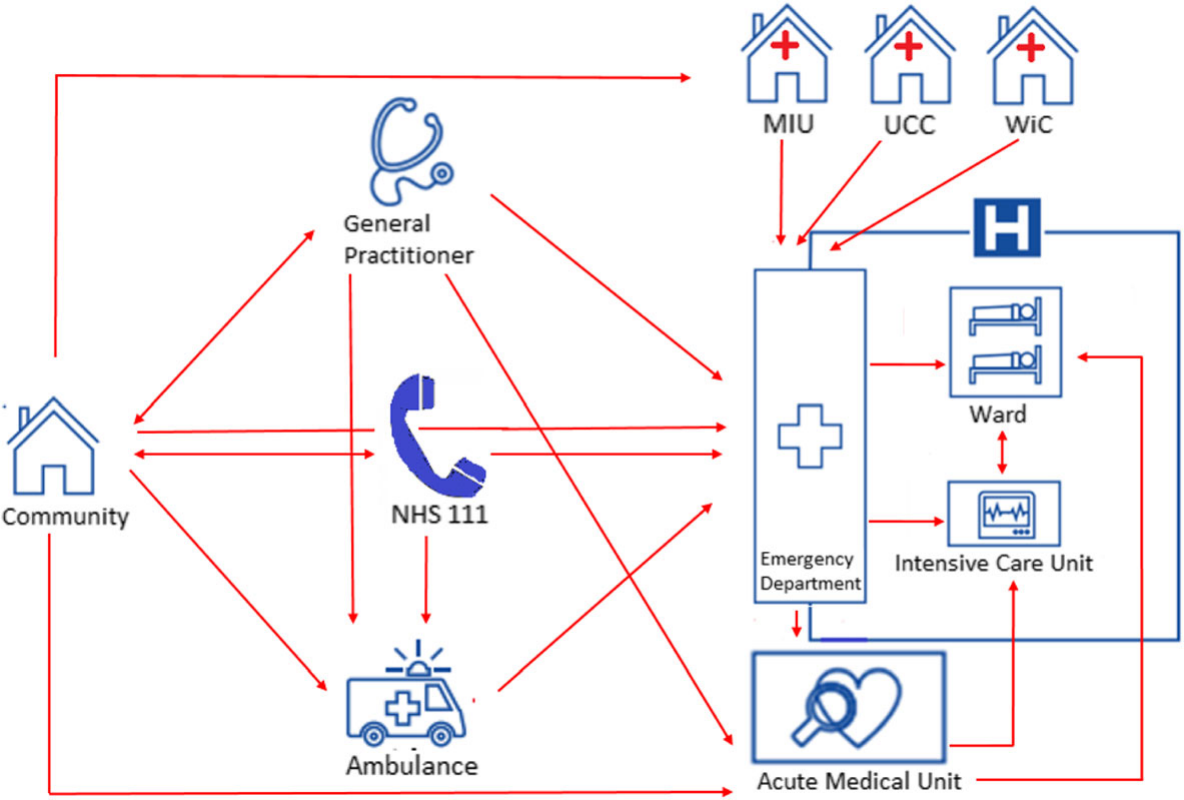
The acute care chain in the UK

In England, acute care has a number of different access points (Fig. 2), which may vary across the different regions. EDs are located within hospitals and the level of service varies: type 1 EDs are major EDs that provide a consultant-led 24-h service with full facilities for resuscitating patients and type 2 EDs are consultant-led facilities but for single specialities. Besides EDs, type 3 departments such as Walk in Centres (WiC), Minor Injury Units (MIU) and Urgent Care Centres (UCC) provide urgent care, treating at least minor injuries or illnesses. These centres are co-located with an ED or sited in the community and can be accessed without an appointment. For completeness in the description of the acute care, Major Trauma Centres function as hubs within a trauma network, mostly placed in larger hospitals. As our analysis focuses only on acute medical care, we have decided to exclude these centres from our overview.

Aiming to guide patients through this system, the NHS provides a telephonic helpline staffed by trained advisers (NHS 111). Depending on the level of perceived urgency these advisors may suggests several alternatives, ranging from self-care to a referral or even sending an ambulance.

In England, there were 7.6 GPs available for a population of 10,000 in 2013. [20] GPs provide urgent care as well, however the accessibility of GP services, particularly out-of-hours and weekend is suboptimal. The most recent NHS GP patient survey showed that 11% of all participants reported not being able to get a timely appointment with their GP. [21] In addition, one publication suggests that a quarter of all self-referred ED visits is due to an unsuccessful attempt to obtain a convenient GP appointment. [22]

At the ED several triage models are in place, differing amongst the UK countries, from which the majority using nurse practitioners to assess patients as they arrive and some of them co-locate GPs at the front door of EDs. Most ED care is provided by emergency physicians and they can decide to admit a patient. Experienced specialty consultants rarely treat patients at the ED.

In 2016 in total 23.4 million ED visits took place in England, of which 32% in type 3 departments. 15.9 million ED visits took place in type 1 and 2 departments, for a population size of 65.6 million, resulting in a 24.2% attendance rate. Since 2007 ED visits have shown a 2.3% increase per year, while the average population growth is 0.8%. 4.3 million patients were admitted acutely in England, which is 27% of all type 1 and 2 department visits and 6.6% of the total population. [23]

This increased demand for emergency care combined with high bed occupancy levels and the increased complexity of care, [24] is a threat to the safety and timeliness of delivering acute care in the UK: 10% of the inspected EDs were rated inadequate on safety and 55% required improvement. [25] Part of the NHS Constitution is the 4 h standard: 95% of patients should be admitted, transferred or discharged within 4 h of arrival at an ED. In March 2018 84.6% of patients were seen within 4 h in all EDs, compared to 90% in March 2017. It’s the lowest performance since introducing this standard. [26]

From the ED, most acute medical patients requiring admission will be admitted to an AMU with exception of acute myocardial infarction and hyperacute stroke patients. The average length of stay in an AMU is < 24 h with a proposed maximum of 48–72 h. [27] During this period patients are discharged directly or transferred to an in-patient specialty ward. AMUs are consultant-led, with a core team of acute physicians supported by specialty physicians. Stable GP referred patients should be admitted directly via the AMU. Another option for stable patients not requiring admission is acute ambulatory care, which can relieve the pressure on EDs and AMUs, although consensus on the level of risk that is appropriate for home-based care has to be reached. Ambulatory care may be provided in 4 different care models: a ‘hospital at home’ setting, quick decision units, out-patient care or observation units. [28]

## Conclusion

Acute medical care in the Netherlands and the UK face similar challenges: an increased pressure on the acute care system due to an increased number of (older) patients attending the ED over the last years, combined with high bed occupancy levels and the increased complexity of care. This leads to an elevated workload, pressure on timeliness and accessibility of acute care and high healthcare costs. In England the number of ED visits per year, per capita is three times higher than in the Netherlands, but the percentage of admissions via type 1 and 2 EDs is 8% lower. However, in England the percentage of acute admissions on a population basis is 1.7% higher than in the Netherlands. Potentially, the triage of patients presenting at the ED in England is less efficient compared to the Netherlands.

The Dutch acute care system finds it strengths in a strong 24/7 primary care system constraining patient flow to the ED. Despite that the concept of first line stays needs further development, it’s a valuable incentive assuring low-complex health care for low-complex cases.

One of the challenges in the Dutch system is the heterogeneously organized emergency care, which makes it hard to establish uniform quality standards for acute care. In addition, differences in organisation can lead to variation in practice, warranted and unwarrented. The staffing of the ED in particular deserves attention, assuring competent and experienced doctors for the care of the most complex patients.

The British acute health care system finds its strengths in the presence of emergency physicians at all EDs. ED physicians can initially see and stabilize patients. Thereafter, acute medical teams operating as part of a multidisciplinary team with specific expertise are needed to treat complex patients who require in-patient care. The organisation of the acute care in the UK is fragmented, leading to ambiguity for patients about which available acute care service they should use. Most important, the increased number of patients visiting the ED combined with high bed occupancy levels and the increased complexity of care, leads to crowding which may potentially affect the timeliness and quality of care.

### Recommendations

By comparing these countries, we believe that lessons in organisation of acute care can be learned from each country. The British organisation of acute care can be improved by strengthening the primary care and community systems by improving access closer to home, increasing the accessibility (24/7) of GPs and optimising use of out of office hours GP services. Introduction of a small fee for patients referring themselves to the ED despite alternative options could help in reducing crowding in EDs, but may be politically hard to achieve. The Dutch ECAP model prevents unnecessary ED visits and reduces the volume pressures in the ED. It is also important that we improve the signposting for urgent care services so patients can more easily navigate the complex system in the UK and access services in a timely manner.

The Dutch system can learn from the British by improving ambulatory care to reduce pressure on in-patient beds and improve patient experience, rather than the more traditional models of out-patient care. Ambulatory emergency care can provide an appropriate support to primary care when escalation is needed, and reduce the use of the inpatient bedbase, thereby facilitating more treatment of acute illnesses from a community setting. [29] In general, The Dutch traditional out-patient care is not focussed on acute illnesses and lacks an adequate availability of ‘acute generalists’ as well as infrastructure facilitating not only a diagnostic, but also a therapeutic response to acutely unwell patients.

Secondly, the roles of emergency physicians and acute physicians should be clear and complementing which may be reached by more uniform staffing. Given the increased complexity of care, experienced consultants need to be present at the ED, providing optimal care pathways, training junior doctors and improving timely and right decision-making and patient flow. It has been shown that presence of consultants at the ED, beside Emergency Physicians, leads to a shorter Length of Stay and higher patient satisfaction. [30]

In both countries the ageing population has led to a changing case mix at the ED with an increased amount of multimorbid patients with polypharmacy. As a result ED presentations are becoming increasingly complex. This requires specialists who are able to deal with these problems, such as internists and geriatricians, and generalists with the ability to coordinate care for these complex patients, such as Emergency Physicians and acute physicians. A way to reach this broader expertise and treat patients in a holistic way, is assuring superspecialism instead of subspecialism for at least internists. Superspecialism requires persisting interest in areas beyond the subspecialty and willingness to practice medicine in a patient-oriented way, in contrast to subspecialism which focusses on a specific area of interest leading to treatment of a disease rather than treating a patient. [31] Therefore, a proportion of all medical specialists should change their attitude and adapt their training and daily practice to superspecialism, which will match the demand of the future case mix. Furthermore, ED-care should be adapted to the elderly: frailty screening, trained medical and para-medical staff, and special care pathways focused on the enablement of the complex needs group of patients may be keys to future-proof acute healthcare.

Finally, to improve the organisation of health care, we believe that doctors need to be visible medical leaders and participate in the organisation of care. Doctors should use their experience and medical knowledge to establish the best acute care working with patients and introduce changes in the organisation in concert with the managers. Medical leadership is considered to play an important role in improving organisational performance, including quality of care, patient safety and cost-efficient care. Furthermore, medical leadership may be necessary to overcome the divide between medical and managerial logics. [32] To assure medical leadership in the future, medical students and residents have to be educated in medical leadership and be shown by role models that leadership will improve the quality of care.

## Data Availability

Data sharing is not applicable to this article as no datasets were generated or analysed during the current study.

## Abbreviations

AMU: Acute Medical Unit
ECAP: Emergency Care Access Point
ED: Emergence Department
GDP: Gross Domestic Product
GP: General Practitioner
MIU: Minor Injury Unit
NHS: National Health Service
UCC: Urgent Care Centre
WiC: Walk in Centre

## References

[1] Zorgautoriteit N (2017). Marktscan acute zorg.

[2] Baker C (2017). Accident and Emergency Statistics: Demand, Performance and Pressure. Briefing, House of Commons Library.

[3] Bernstein SL, Aronsky D, Duseja R (2009). The effect of emergency department crowding on clinically oriented outcomes. Acad Emerg Med.

[4] Brouwers C, Merten H, Willems M (2017). Improving care for older patients in the acute setting: a qualitative study with healthcare providers. Neth J Med.

[5] George G, Jell C, Todd BS (2006). Effect on population ageing on emergency department speed and efficiency: a historical perspective from a district hospital in the UK. Emerg Med J.

[6] Schrijver EJ, Toppinga Q, de Vries OJ (2013). An observational cohort study on geriatric patient profile in an emergency department in the Netherlands. Neth J Med.

[7] Royal College of Emergency Medicine. *Tackling emergency department crowding.* Retrieved from: https://www.rcem.ac.uk/docs/College%20Guidelines/5z23.%20ED%20crowding%20overview%20and%20toolkit%20(Dec%202015).pdf. Accessed 24 July 2018.

[8] Hoot NR, Aronsky D (2008). Systematic review of emergency department crowding: causes, effects, and solutions. Ann Emerg Med.

[9] Jayaprakash N, O’Sullivan R, Bey T (2009). Crowding and delivery of healthcare in Emergency Departments: the European perspective. Western J Emerg Med.

[10] Baier N, Geissler A, Bech M (2018). Emergency and urgent care systems in Australia, Denmark, England, France, Germany and the Netherlands – Analyzing organisation, payments and reforms. Health Policy.

[11] Kingsfund. *What’s going on with A&E waiting times?* https://www.kingsfund.org.uk/projects/urgent-emergency-care/urgent-and-emergency-care-mythbusters. Accessed 15 Apr 2019.

[12] Staat volksgezondheid en zorg. *Ziekenhuisbedden*. https://www.staatvenz.nl/kerncijfers/ziekenhuisbedden. Accessed 15 Apr 2019.

[13] Eurostat. *Healthcare expenditure statistics.* Retrieved from: http://ec.europa.eu/eurostat/statistics-explained/index.php/Healthcare_expenditure_statistics. Accessed 28 Aug 2018.

[14] Van der Velden LFJ, Kasteleijn A, Kenens RJ. Cijfers uit de registratie van huisartsen; peiling 2016. Retrieved from: https://nvl004.nivel.nl/nivel-2015/sites/default/files/cijfers-uit-de-registratie-van-huisartsen-peiling-januari-2016.pdf. Accessed 18 Apr 2019.

[15] Jansen T, Smits M, Verheij R. *Zorg op de huisartsenpost - Triage.* From: NIVEL Zorgregistraties eerste lijn. Retrieved from: https://www.nivel.nl/nl/nivel-zorgregistraties-eerste-lijn/triage. Accessed 28 Aug 2018.

[16] Smits M, Rutten M, Keizer E (2017). The development and performance of After-Hours primary care in the Netherlands. Ann Intern Med.

[17] Landelijke Huisartsen Vereniging. *Eerstelijnsbedden: snelle oplossing voor patiënten vaak niet gevonden.* https://www.lhv.nl/actueel/nieuws/eerstelijnsbedden-snelle-oplossing-voor-patienten-vaak-niet-gevonden. Accessed 29 Apr 2019.

[18] Fluent. *Onderzoek naar duurzame inrichting spoedzorg keten voor ouderen.* http://fluent.nl/wp-content/uploads/2018/02/ActiZ_onderzoek-naar-duurzame-inrichting-spoedzorgketen-voor-ouderen_door-fluent_5-feb-2018_DEF.pdf. Accessed 29 Apr 2019.

[19] Centraal Bureau voor de Statistiek. *Bevolking; kerncijfers.* https://statline.cbs.nl/Statweb/publication/?DM=SLNL&PA=37296ned&D1=a&D2=0,10,20,30,40,50,63-66&HDR=G1&STB=T&VW=T. Accessed 29 Apr 2019.

[20] Local Government Association. *Ratio of GPs per 10,000 population in England.* https://lginform.local.gov.uk/reports/lgastandard?mod-metric=3713&mod-area=E92000001&mod-group=AllRegions_England&mod-type=namedComparisonGroup. Accessed 27 Apr 2019.

[21] NHS England. *Improving access to general practice.* Retrieved from: https://www.england.nhs.uk/wp-content/uploads/2017/11/improving-access-general-practice-national-slidedeck.pdf. Accessed 27 Apr 2019.

[22] Cowling TE, Harris MJ, Watt HC (2014). Access to general practice and visits to accident and emergency (A&E) departments in England: cross-sectional analysis of a national patient survey. Br J Gen Pract.

[23] NHS England and NHS Digital. Hospital Accident and Emergency activity 2016-2017*.* Retrieved from: https://files.digital.nhs.uk/pdf/m/4/acci-emer-atte-eng-2016-17-rep.pdf. Accessed 24 July 2018.

[24] Kingsfund. *NHS hospital bed numbers: past, present, future.* https://www.kingsfund.org.uk/publications/nhs-hospital-bed-numbers. Accessed 27 Apr 2019.

[25] Care Quality Commission. (Accessed Apr 2018) The state of care in NHS acute hospitals: 2014 to 2016. Retrieved from: www.cqc.org.uk/sites/default/files/20170302b_stateofhospitals_web.pdf. Accessed 12 Apr 2018.

[26] NHS (2018). A&E attendances and emergency admissions, statistical commentary.

[27] Acute Medicine Task Force (2007). Acute Medical care: the right person, in the right setting – the first time. Report.

[28] Lasserson DS, Harris C, Elias T (2018). What is the evidence base for ambulatory care for acute medical illness?. Acute Med.

[29] Cottrel E, Mallen CD, Lasserson DS (2018). Ambulatory emergency care: how should acute generalists manage risk in undifferentiated illness?. Br J Gen Pract.

[30] Van der Linden MC, de Beaufort RAY, Meylaerts SAG (2019). The impact of medical specialist staffing on emergency department flow and satisfaction. Eur J Emerg Med.

[31] Levi M (2017). Generalism in modern subspecializing medicine. Eur J Intern Med.

[32] Berghout Mathilde A., Fabbricotti Isabelle N., Buljac-Samardžić Martina, Hilders Carina G. J. M. (2017). Medical leaders or masters?—A systematic review of medical leadership in hospital settings. PLOS ONE.

